# Exploring Knowledge, Awareness, and Practices Regarding Periodontal Health Assessment and Mechanical Plaque Control Among the Shillong Population of Meghalaya, India: A Descriptive Cross-Sectional Investigation

**DOI:** 10.7759/cureus.57692

**Published:** 2024-04-05

**Authors:** Saismita Sahoo, Dhirendra Kumar Singh, Naina Pattnaik, Mohammad Jalaluddin, Debasish Mishra, Arpita Mohapatra, Jugajyoti Pati

**Affiliations:** 1 Department of Periodontology, Kalinga Institute of Dental Sciences, Kalinga Institute of Industrial Technology Deemed to be University, Bhubaneswar, IND; 2 Department of Oral and Maxillofacial Surgery, Kalinga Institute of Dental Sciences, Kalinga Institute of Industrial Technology Deemed to be University, Bhubaneswar, IND

**Keywords:** toothbrush, oral health, awareness, knowledge, shillong

## Abstract

Background

Periodontal diseases are widespread oral health conditions. However, there remains a lack of comprehensive understanding regarding the knowledge, awareness, and practices related to periodontal health assessment and mechanical plaque control among specific populations, such as those residing in Shillong, Meghalaya. Shillong, being the capital city of Meghalaya in northeastern India, represents a diverse demographic and cultural landscape.

Aim

This study aims to evaluate the knowledge, awareness, and practices related to mechanical plaque control among the population of Shillong City.

Methodology

A descriptive cross-sectional online survey was conducted among the residents of Shillong City, Meghalaya. Data collection involved the administration of an 18-item, closed-ended, self-structured questionnaire. Before the main data collection, a pilot study was conducted involving 63 individuals. Data analysis was performed using IBM SPSS Statistics for Windows, Version 26.0 (Released 2019; IBM Corp., Armonk, NY, USA), employing the chi-square test and ANOVA with a significance level of 0.05.

Results

Study participants were categorized into five age groups spanning from 21 to 64 years old, with the age group of 41 to 50 years demonstrating the highest mean knowledge score. Age exhibited a statistically significant influence on knowledge scores.

Conclusion

The study reveals a commendable level of knowledge, awareness, and adherence to practices regarding the primary tool for oral hygiene maintenance, the toothbrush, among the residents of Shillong City.

## Introduction

The escalating population growth in India is accompanied by a corresponding increase in the incidence of various diseases, among which periodontal diseases are prevalent, ranging from 50% to 100% across different regions of the Indian subcontinent [1]. Periodontal disease stands as one of the foremost dental conditions affecting adults, representing a significant chronic inflammatory ailment affecting a substantial segment of the populace.

Periodontal diseases, encompassing gingivitis and periodontitis, can exert their effects on individual teeth or multiple teeth, potentially leading to tooth loss if left untreated [2]. Hence, timely intervention for periodontal issues is imperative to safeguard dental integrity and overall oral health [3]. The etiology of periodontitis is multifactorial, with poor oral hygiene and addictive substance habits being prime contributors [4]. Of particular concern is the widespread consumption of betel (*Areca*) nuts, a prevalent oral chewing habit globally that has been associated with the development of oral cancer. India grapples with an alarming surge in registered oral cancer cases, particularly concentrated in hotspots like the northeastern states of Assam and Meghalaya [1,5].

The onset of addictive habits often begins at an early age, warranting urgent intervention strategies to deter addiction and prioritize oral hygiene, especially in regions like Meghalaya where addictive substance usage is more prevalent [6,7]. Prevention of periodontal diseases hinges on effective mechanical and chemical plaque control, necessitating correct periodontal assessment, appropriate tooth brushing and flossing techniques, routine dental checkups, and adherence to suitable dietary practices [8,9]. The most effective strategy for preventing periodontal diseases unequivocally lies in the mechanical approach to plaque reduction. While toothbrushing effectively combats gingivitis on buccal and lingual surfaces, its efficacy in interdental spaces is limited [10]. Consequently, supplementary materials, such as interdental floss and oral irrigators, have been developed to address this lacuna.

Although the global recommendation for using dental floss as an adjunct to oral hygiene varies, its effectiveness in removing interdental plaque surpasses that of a manual toothbrush alone [11]. However, reports indicate that only a minority of individuals utilize dental floss. Despite mechanical plaque removal remaining pivotal in managing periodontal diseases, awareness of its significance remains deficient [12]. Dentists play an integral role in patient education and fostering awareness about preventive measures for periodontal diseases and overall oral health.

To this end, the present study focuses on a cross-section of Shillong, where addictive habits are prevalent, aiming to assess the knowledge, awareness, and practice of mechanical plaque control among the populace.

## Materials and methods

Study population and sample size

A cross-sectional online survey was conducted among the study population in Shillong, spanning from November 2023 to February 2024. The survey employed a purposive sampling method, ensuring representation across different age groups, genders, and socioeconomic backgrounds, and the sample size comprised 305 participants who provided informed consent. The ethical clearance for the study was obtained from the Institutional Ethical Committee of Kalinga Institute of Dental Sciences (approval number KIDS/RES/036/2023).

The sample size was calculated based on the following formula: sample size: n = Z^2^ p (1-p)/d^2^, where z = standardized normal deviation (z value) of 1.96 for 95%, p = prevalence of interest, and d = clinically expected variation of 10% of the prevalence of interest.

Inclusion and exclusion criteria

Eligible participants for the study were individuals residing in Shillong who possessed smartphones with internet connectivity. The inclusion criteria stipulated that participants must be over 18 years of age. Exclusion criteria encompassed individuals who declined to provide informed consent, lacked a permanent address within the selected states, or did not possess cell phones.

Data collection

A closed-ended, self-structured questionnaire consisting of 18 items was developed to collect data for the study. The questionnaire was divided into four sections, starting with sociodemographic information, followed by inquiries regarding knowledge, awareness, and practices concerning mechanical plaque control. Before the main data collection, a pilot study involving 63 individuals was conducted, which was not included in the final sample size. The questionnaire was validated by three specialists and a statistician from the Department of Periodontics and Implantology at the Kalinga Institute of Industrial Technology Deemed to be University, with a calculated Cronbach’s alpha of 0.85.

The finalized questionnaire was disseminated to the target population using Google Forms (Google LLC, Mountain View, California, United States) via WhatsApp (WhatsApp LLC, Menlo Park, California, United States) and email platforms for known contacts. Informed consent was obtained from all participants. Four incomplete surveys were received and subsequently excluded from the analysis, resulting in a total sample size of 301 individuals.

Statistical analysis

The collected data were imported into Microsoft Excel (Microsoft Corporation, Redmond, Washington, United States) and subjected to analysis using IBM SPSS Statistics for Windows, Version 26.0 (Released 2019; IBM Corp., Armonk, NY, USA). Descriptive statistics, such as frequencies and percentages, were utilized to characterize categorical variables. Statistical tests, including the chi-square test and ANOVA, were employed to assess relationships and differences within the data. A significance level of 0.05 was predetermined for all analyses.

## Results

In this study, participants were categorized into five age groups spanning from 21 to 64 years old, with 52.8% identified as male and 47.2% as female (Table 1).

**Table 1:** Sociodemographic data

Variable	Category	Frequency	Percent
Age	20-30	163	54.2
	31-40	64	21.3
	41-50	25	8.3
	51-60	25	8.3
	60 above	24	8.0
Gender	Male	159	52.8
	Female	142	47.2

Descriptive statistics for the 18-item questionnaire and participants’ responses are presented in Table 2.

**Table 2:** Responses of study participants (n = 301)

Domain	Question	Option	Frequency	Percentage
Knowledge	K1: Best way for plaque control	Brushing	99	32.9
		Toothpick	5	1.7
		Mouthwash	9	3.0
		All	188	62.5
	K2: What comes under chemical plaque control?	Saline	40	13.3
		Mouthwash	69	22.9
		Bleaching	34	11.3
		Clove oil	10	3.3
		All	148	49.2
	K3: What comes under mechanical control?	Toothbrush	54	17.9
		Dental floss	20	6.6
		Interdental brush	39	13.0
		Powered toothbrush	10	3.3
		All	178	59.1
	K4: Brushing more than twice daily vigorously with a hard bristle brush helps in plaque control	No	147	48.8
		Yes	74	24.6
		Maybe	80	26.6
	K5: A sonic powered toothbrush helps reduce plaque formation and deposition in the oral cavity	No	25	8.3
		Yes	95	31.6
		Maybe	181	60.1
	K6: Most effective in maintaining plaque or interdental cleaning	Dental floss	53	17.6
		Interdental brush	5	1.7
		Single tufted brush or rubber tip	5	1.7
		Toothbrush	49	16.3
		All of the above	189	62.8
Awareness	A1: Aware of plaque present in our oral cavity	No	35	11.6
		Yes	218	72.4
		Maybe	48	15.9
	A2: Accumulation of plaque affects oral health and the whole body	No	10	3.3
		Yes	233	77.4
		Maybe	58	19.3
	A3: Heard of mechanical plaque control	No	124	41.2
		Yes	84	27.9
		Maybe	93	30.9
	A4: If you know about mechanical plaque control, then from where did you get the acknowledgment of it?	Advertisements	78	25.9
		Social media	60	19.9
		Television	75	24.9
		From friends	78	25.9
		Print media	10	3.3
	A5: Heard of dental floss	No	44	14.6
		Yes	233	77.4
		Maybe	24	8.0
	A6: Heard of interdental brushes	No	165	54.8
		Yes	89	29.6
		Maybe	47	15.6
Practice	P1: Form of mechanical plaque control you use	Toothbrush	238	79.1
		Dental floss	19	6.3
		Interdental brush	5	1.7
		All	39	13.0
	P2: Use all the mechanical plaque control aids	No	35	11.6
		Yes	177	58.8
		Maybe	89	29.6
	P3: Will you suggest your near and dear ones use mechanical plaque control aids?	No	20	6.6
		Yes	246	81.7
		Maybe	35	11.6
	P4: Use dental floss in your daily oral hygiene maintenance	No	138	45.8
		Yes	129	42.9
		Maybe	34	11.3
	P5: Use wood sticks quite often	No	169	56.1
		Yes	93	30.9
		Maybe	39	13.0
	P6: Use a single tufted brush	No	197	65.4
		Yes	50	16.6
		Maybe	54	17.9

Analysis of knowledge, awareness, and practice domain scores revealed variations across age groups. Notably, individuals aged 41 to 50 years demonstrated the highest mean knowledge score of 3.40 ± 1.384, with age exerting a statistically significant influence on knowledge scores. Additionally, significant differences were observed among participant groups concerning awareness and practices in items A1-A5 and P1-P5 (Table 3).

**Table 3:** Knowledge, awareness, and practice of study participants according to gender * indicates a significant difference at p ≤ 0.05

Domain		Age	Mean	Standard deviation	Standard error	95% CI for mean		F	p-value
						Lower bound	Upper bound		
Knowledge	K1-K6	20-30	3.29	1.474	0.115	3.07	3.52	6.524	<0.001*
		31-40	3.34	1.312	0.164	3.02	3.67		
		41-50	3.40	1.384	0.277	2.83	3.97		
		51-60	1.80	1.979	0.396	0.98	2.62		
		60 above	2.67	1.659	0.339	1.97	3.37		
Awareness	A1	20-30	1.15	0.500	0.039	1.07	1.22	19.333	<0.001*
		31-40	1.22	0.417	0.052	1.11	1.32		
		41-50	0.80	0.408	0.082	0.63	0.97		
		51-60	0.40	0.500	0.100	0.19	0.61		
		60 above	0.79	0.415	0.085	0.62	0.97		
	A2	20-30	1.27	0.445	0.035	1.20	1.34	17.022	<0.001*
		31-40	1.22	0.417	0.052	1.11	1.32		
		41-50	1.00	0.000	0.000	1.00	1.00		
		51-60	0.60	0.500	0.100	0.39	0.81		
		60 above	1.00	0.000	0.000	1.00	1.00		
	A3	20-30	1.03	0.871	0.068	0.90	1.17	2.496	0.043*
		31-40	0.77	0.707	0.088	0.59	0.94		
		41-50	0.60	0.816	0.163	0.26	0.94		
		51-60	0.80	1.000	0.200	0.39	1.21		
		60 above	0.75	0.737	0.150	0.44	1.06		
	A4	20-30	2.38	1.177	0.092	2.20	2.56	19.870	<0.001*
		31-40	2.59	1.003	0.125	2.34	2.84		
		41-50	3.80	1.000	0.200	3.39	4.21		
		51-60	1.80	1.000	0.200	1.39	2.21		
		60 above	3.79	0.779	0.159	3.46	4.12		
	A5	20-30	1.01	0.423	0.033	0.94	1.07	5.657	<0.001*
		31-40	0.98	0.378	0.047	0.89	1.08		
		41-50	0.80	0.408	0.082	0.63	0.97		
		51-60	0.60	0.816	0.163	0.26	0.94		
		60 above	0.79	0.415	0.085	0.62	0.97		
	A6	20-30	0.66	0.723	0.057	0.54	0.77	1.384	0.239
		31-40	0.59	0.830	0.104	0.39	0.80		
		41-50	0.40	0.500	0.100	0.19	0.61		
		51-60	0.40	0.816	0.163	0.06	0.74		
		60 above	0.75	0.737	0.150	0.44	1.06		
Practice	P1	20-30	1.65	1.417	0.111	1.43	1.87	2.690	0.031*
		31-40	1.63	1.464	0.183	1.26	1.99		
		41-50	1.40	0.816	0.163	1.06	1.74		
		51-60	1.00	0.000	0.000	1.00	1.00		
		60 above	2.21	1.532	0.313	1.56	2.86		
	P2	20-30	1.15	0.558	0.044	1.06	1.23	4.289	0.002*
		31-40	1.31	0.614	0.077	1.16	1.47		
		41-50	1.20	0.764	0.153	0.88	1.52		
		51-60	0.80	0.764	0.153	0.48	1.12		
		60 above	1.42	0.504	0.103	1.20	1.63		
	P3	20-30	0.94	0.346	0.027	0.89	0.99	9.938	<0.001*
		31-40	1.08	0.270	0.034	1.01	1.15		
		41-50	1.20	0.408	0.082	1.03	1.37		
		51-60	1.20	0.764	0.153	0.88	1.52		
		60 above	1.42	0.504	0.103	1.20	1.63		
	P4	20-30	0.66	0.722	0.057	0.55	0.77	3.850	0.005*
		31-40	0.78	0.701	0.088	0.61	0.96		
		41-50	0.60	0.500	0.100	0.39	0.81		
		51-60	0.20	0.408	0.082	0.03	0.37		
		60 above	0.79	0.415	0.085	0.62	0.97		
	P5	20-30	0.66	0.765	0.060	0.54	0.77	10.603	0.001*
		31-40	0.16	0.366	0.046	0.06	0.25		
		41-50	0.80	0.764	0.153	0.48	1.12		
		51-60	1.00	0.645	0.129	0.73	1.27		
		60 above	0.38	0.495	0.101	0.17	0.58		
	P6	20-30	0.57	0.853	0.067	0.44	0.70	0.637	0.637
		31-40	0.47	0.755	0.094	0.28	0.66		
		41-50	0.40	0.500	0.100	0.19	0.61		
		51-60	0.40	0.816	0.163	0.06	0.74		
		60 above	0.63	0.495	0.101	0.42	0.83		

Furthermore, a comparative assessment of domain scores across gender groups revealed significant differences in all knowledge items except K3 (Table 4). Similarly, items A2, A3, and A5 displayed statistical significance in awareness, while all items except P3 exhibited significance in practice.

**Table 4:** Knowledge, awareness, and practices of study participants according to gender * indicates a significant difference at p ≤ 0.05

Domain	Question	Option	Male, n (%)	Female, n (%)	p-value
Knowledge	K1	Brushing	55 (55.6)	44 (44.4)	0.002*
		Toothpick	5 (100)	0	
		Mouthwash	9 (100)	0	
		All	90 (47.9)	98 (52.1)	
	K2	Saline	20 (50)	20 (50)	0.022*
		Mouthwash	25 (36.2)	44 (63.8)	
		Bleaching	20 (58.8)	14 (41.2)	
		Clove oil	5 (50)	5 (50)	
		All	89 (60.1)	59 (39.9)	
	K3	Toothbrush	35 (64.8)	19 (35.2)	
		Dental floss	15 (75)	5 (25)	0.056
		Interdental brush	19 (48.7)	20 (51.3)	
		Powered toothbrush	5 (50)	5 (50)	
		All	85 (47.8)	93 (52.2)	
	K4	No	50 (34)	97 (66)	0.0001*
		Yes	54 (73)	20 (27)	
		Maybe	55 (68.8)	25 (31.3)	
	K5	No	10 (40)	15 (60)	0.001*
		Yes	65 (68.4)	30 (31.6)	
		Maybe	84 (46.4)	97 (53.6)	
	K6	Dental floss	25 (47.2)	28 (52.8)	
		Interdental brush	0	5	0.002*
		Single tufted brush or rubber tip	5 (100)	0	
		Toothbrush	34 (69.4)	15 (30.6)	
		All of the above	95 (50.3)	94 (49.7)	
Awareness	A1	No	20 (57.1)	15 (42.9)	0.862
		Yes	114 (52.3)	104 (47.7)	
		Maybe	25 (52.1)	23 (47.9)	
	A2	No	10 (100)	0	0.004*
		Yes	124 (53.2)	109 (46.8)	
		Maybe	25 (43.1)	33 (56.9)	
	A3	No	75 (60.5)	49 (39.5)	0.001*
		Yes	50 (59.5)	34 (40.5)	
		Maybe	34 (36.6)	59 (63.4)	
	A4	No	30 (68.2)	14 (31.8)	0.039
		Yes	114 (48.9)	119 (51.1)	
		Maybe	15 (62.5)	9 (37.5)	
	A5	Advertisements	25 (32.1)	53 (67.9)	0.001*
		Social media	35 (58.3)	25 (41.7)	
		Television	50 (66.7)	25 (33.3)	
		From friends	39 (50)	39 (50)	
		Print media	10 (100)	0	
	A6	No	95 (57.6)	70 (42.4)	0.101
		Yes	45 (50.6)	44 (49.4)	
		Maybe	19 (40.4)	28 (59.6)	
Practice	P1	Toothbrush	110 (46.2)	128 (53.8)	0.0001*
		Dental floss	19 (100)	0	
		Interdental brush	0	5 (100)	
		All	30 (76.9)	9 (23.1)	
	P2	No	20 (57.1)	15 (42.9)	0.0001*
		Yes	74 (41.8)	103 (58.2)	
		Maybe	65 (73)	24 (27)	
	P3	No	10 (50)	10 (50)	0.064
		Yes	124 (50.4)	122 (49.6)	
		Maybe	25 (71.4)	10 (28.6)	
	P4	No	60 (43.5)	78 (56.5)	0.011*
		Yes	79 (61.2)	50 (38.8)	
		Maybe	20 (58.8)	14 (41.2)	
	P5	No	80 (47.3)	89 (52.7)	0.043*
		Yes	59 (63.4)	34 (36.6)	
		Maybe	20 (51.3)	19 (48.7)	
	P6	No	94 (47.7)	103 (52.3)	0.045*
		Yes	30 (60)	20 (40)	
		Maybe	35 (64.8)	19 (35.2)	

## Discussion

In 2016, the inaugural endeavor to evaluate India’s state-wise Global Disease Burden omitted oral health, an essential component of overall well-being. Instead, this study relied on prevalence data concerning oral diseases spanning from 2001 to 2004 [13].

Periodontitis, a concerning condition, stems from various etiological factors, including inadequate oral hygiene and addictive habits.

Moreover, the widespread consumption of betel nuts, a prevalent oral habit globally, has been implicated in oral cancer development, with India bearing the highest burden of registered cases worldwide. Predominantly, betel nut consumption is concentrated in India’s North Eastern Region, coastal areas, and select parts of the northern plains [1,14].

The concept of “periodontal medicine,” coined by Offenbacher, encompasses a burgeoning sub-specialty of periodontology that underscores the interplay between periodontal health and systemic well-being [15]. This paradigm shift acknowledges bidirectional influences, wherein periodontal health can impact systemic health and vice versa, a relationship that has long been observed alongside traditional dentistry [16].

Fundamental oral health knowledge is pivotal for fostering self-preventive behaviors and adopting effective preventive measures. This includes consistent dental hygiene practices, dietary modifications, and adherence to professional guidance and care [17]. Notably, the reduction of plaque formation plays a pivotal role in mitigating the prevalence of dental caries, gingivitis, and periodontal diseases [18,19].

Gender emerges as a consistent determinant of tooth brushing frequency, with females typically exhibiting greater concern for personal hygiene compared to males. This gender disparity underscores the importance of tailored oral health interventions.

A study conducted by Aryal et al. to evaluate the knowledge, attitude, and practices concerning periodontal health among patients visiting a dental college demonstrated good oral hygiene practices among the patients with favorable knowledge and attitude concerning periodontal health and its measures to control the disease [20]. A study was conducted by Suragimath et al. to assess the knowledge, attitude, and practices concerning periodontal diseases among school teachers. The results showed that the majority of the teachers had good knowledge about the causes and prevention of gingival diseases, but awareness and practices were poor [21]. A cross-sectional study conducted by Cinthya et al. to evaluate the oral health knowledge, attitudes, and behavior of the patients showed a lack of awareness and negligence toward oral health among the general public [22]. A study conducted by Kannan et al. among children to assess their awareness of mechanical and chemical plaque control showed that 72% of the population brushes their teeth two times per day [23].

Mechanical plaque control, encompassing knowledge, attitudes, and practices, is indispensable for both personal oral hygiene and patient education [24]. Dentists and healthcare professionals play a crucial role in imparting education and motivating patients to adhere to optimal oral hygiene practices, including interdental cleaning procedures [25]. This collaborative approach aligns with the evolving paradigm of periodontal medicine, wherein oral health becomes increasingly intertwined with overall health outcomes. Effective communication between dental and medical practitioners is essential, necessitating a deeper understanding of systemic disorders among dental professionals and the integration of new educational objectives within the profession.

## Conclusions

In Shillong, there exists a commendable level of knowledge, awareness, and adherence to practices concerning the primary tool for oral hygiene maintenance, namely the toothbrush. However, the utilization of interdental aids to enhance oral hygiene practices among the populace is notably low. Further investigation is warranted to assess the oral health and periodontal status of individuals employing solely toothbrushes versus those incorporating interdental devices into their oral care regimen. Such research endeavors would elucidate the efficacy and routine recommendation of interdental assistance.

In the Indian context, enhancing periodontal knowledge by promoting the utilization of interdental devices alongside toothbrushing represents a pivotal public health imperative. Emphasizing the intrinsic link between dental health and overall bodily well-being is paramount.
